# 3D Hierarchical, Nanostructured Chitosan/PLA/HA Scaffolds Doped with TiO_2_/Au/Pt NPs with Tunable Properties for Guided Bone Tissue Engineering

**DOI:** 10.3390/polym12040792

**Published:** 2020-04-02

**Authors:** Julia Radwan-Pragłowska, Łukasz Janus, Marek Piątkowski, Dariusz Bogdał, Dalibor Matysek

**Affiliations:** 1 Department of Physical Chemistry, Faculty of Chemical Engineering and Technology, Cracow University of Technology, 31–155 Cracow, Poland; j.radwan@doktorant.pk.edu.pl (J.R.-P.); lukasz.janus@doktorant.pk.edu.pl (Ł.J.); pcbogdal@cyf-kr.edu.pl (D.B.); 2 Faculty of Mining and Geology, Technical University of Ostrava; 708 00 Ostrava, Czech Republic; dalibor.matysek@vsb.cz

**Keywords:** smart hybrid materials, properties of nanoparticles–reinforced polymers, biotechnology

## Abstract

Bone tissue is the second tissue to be replaced. Annually, over four million surgical treatments are performed. Tissue engineering constitutes an alternative to autologous grafts. Its application requires three-dimensional scaffolds, which mimic human body environment. Bone tissue has a highly organized structure and contains mostly inorganic components. The scaffolds of the latest generation should not only be biocompatible but also promote osteoconduction. Poly (lactic acid) nanofibers are commonly used for this purpose; however, they lack bioactivity and do not provide good cell adhesion. Chitosan is a commonly used biopolymer which positively affects osteoblasts’ behavior. The aim of this article was to prepare novel hybrid 3D scaffolds containing nanohydroxyapatite capable of cell-response stimulation. The matrixes were successfully obtained by PLA electrospinning and microwave-assisted chitosan crosslinking, followed by doping with three types of metallic nanoparticles (Au, Pt, and TiO_2_). The products and semi-components were characterized over their physicochemical properties, such as chemical structure, crystallinity, and swelling degree. Nanoparticles’ and ready biomaterials’ morphologies were investigated by SEM and TEM methods. Finally, the scaffolds were studied over bioactivity on MG-63 and effect on current-stimulated biomineralization. Obtained results confirmed preparation of tunable biomimicking matrixes which may be used as a promising tool for bone-tissue engineering.

## 1. Introduction

Bone is one of the tissues with the ability of self-regeneration and constant remodeling [1,2]. Despite this fact, each year, over four million surgeries are performed in order to treat this tissue’s defects, using autografts or bone substitutes [1,2]. The history of bone pathologies which cannot be self-healed is very long and concerns both congenital and acquired ones. These include traumas, neoplasm, infection or failed arthroplasty, spine arthrodesis, implant fixation, and others [2]. Because of that, bone is known to be the second most commonly replaced tissue [1,2]. Since the application of a patient’s own bone is limited by the size of the defect, bone substitutes are rapidly gaining attention. Grafts coming from other donors, so called allografts, carry the risk of transmitting disease, infection, and its rejection. Alternatively, used metal (stainless steel, pure titanium, or its alloys) and synthetic biomaterials have some major limitations, such as poor biocompatibility, biocorrosion, and consumption over time. Non-invasive methods can only accelerate the healing process [2]. To overcome this issues, novel types of biomaterials are developed which mimic natural bone composition and structure [1,2,3,4].

Tissue engineering is one of the most powerful tools used in medicine which brings hope to the millions of patients suffering from various dysfunctions caused by diseases, traumas, or injuries. Its main objective is to restore, improve, or maintain biological functions of damaged tissue [5]. This strategy involves application of the mechanical support called scaffold, which also provides biological help to the cells, due to its chemical composition. To improve their properties, scaffolds can be prepared from various raw materials and are often functionalized with bioactive molecules, such as growth factors or antibiotics [1,2,3,5].

The biomaterials for bone regeneration are prepared in the form of three-dimensional spatial structure which mimics natural tissue by various methods. Porous materials can be embedded with various substances which promote osteoconduction. The ideal scaffold for this application should meet five conditions, namely biocompatibility, biodegradability, bioactivity, appropriate architecture, and high durability [4,5,6,7,8].

Scaffold dedicated to bone-tissue engineering should provide a temporary mechanical support for the damaged area and stimulate tissue regeneration [2,9]. Therefore, it must be characterized by highly porous architecture, to enable bone ingrowth, as well as neovascularization. The scaffold should constitute a template which promotes extracellular matrix formation and a place for osteoblasts to adhere and proliferate. Such biomaterials can have a form of nanofibers, hydrogels, metal alloys, β-TCP, HA powders, and granules or bioactive glasses [10,11]. Among them, the most promising ones are nanofibrous scaffolds, which can be prepared through electrospinning of the polymer solution since they biomimic natural ECM architecture [12,13,14,15,16]. Their properties, such as high surface area and porosity make them a highly desired materials in terms of bone-tissue-defect treatment [17,18,19]. However, due to the hierarchical nature of the bone, nanofibrous/porous composites seem to be the most promising ones [5,9].

Scaffolds for bone-tissue engineering that are prepared from minerals have poor mechanical durability, but at the same time, they exhibit good biocompatibility and the potential for various molecules’ release. Natural polymers undergoing enzymatic biodegradation which speed rate may be adjusted are used as scaffold components and drug-delivery and -release systems [20]. The most common ones are collagen, fibrin, gelatin, chitin, and its derivatives, or alginate; additionally, biodegradable polymers, such as PLGA/PLA and PCL are widely used [21,22,23,24]. Notably, their low durability and fast degradability means that they must be used in conjunction with other materials [14,15,16]. Electrospinning enables preparation of micro- and nanofibers, using both raw polymers solution as well as composites and nanocomposites [19]. To enhance NFs properties, ready products may undergo postprocessing functionalization by various substances, such as hydroxyapatite, β-TCP, metal, or metal oxide nanoparticles [5,9,10,25,26,27]. Such an approach enables preparation of bone-tissue scaffolds with tunable properties, including programmed biodegradability rate, enhanced mechanical durability, crystallinity, antibacterial, bioactivity, and others [25,28]. There are some synthetic polymers approved by the FDA, such as polycaprolactone (PCL) and polylactic acid PLA, which can be successfully used for nanofibers’ preparation. Other polymers include polyglycolic Acid (PGA) and polyethylene glycol (PEG). Nevertheless, their degradation products cause a local pH decrease due to their acidic nature, which negatively affects proteins and trigger inflammation [1,2,3,4,5]. Chitosan is a poly(saccharide) which is used for porous sponges and nanofibers preparation, yet due to its problems associated with solubility in different solvents, this biopolymer application requires chemical or physical modification [20,21,22,23,24].

The aim of the following research was to develop a novel hybrid material of hierarchical structure with enhanced biological properties. Prepared three-dimensional biomaterials for bone-tissue engineering were developed based on the application of Au [29,30], Pt [31], and TiO_2_ [32,33,34,35] nanoparticles embedded on PLA nanofibers functionalized with hydroxyapatite and highly porous chitosan aerogel matrix. The addition of the nanoparticles has a positive impact on the mechanical properties of the scaffolds, and due to their ability of current conductivity, they enable cell-proliferation stimulation by direct current [25,26,27,28]. Moreover, the presence of the NPs has a positive impact on various cell responses by itself [25,26,27]. The nanoparticles can also help to reduce the risk of bacterial infections, since they exhibit antibacterial activity against various bacterial strains, such as *S. aureus* or *E. coli*. The bactericidal effect occurs due to genetic material damage or disruption of cell membrane [25,26,27,28]. The composites were prepared by using polymers of known applicability in tissue engineering [36,37,38]. Ready scaffolds with tunable properties were characterized over their physicochemical properties, such as chemical structure, crystallinity, morphology, effect on biomineralization, and biocompatibility with human osteosarcoma (MG-63) cell lines [39,40,41]. Obtained results showed that proposed biomaterials display extraordinary properties and may significantly contribute to bone-tissue-engineering development.

## 2. Materials and Methods

### 2.1. Materials

Fungal chitosan (300,000 g/mol) and 85% deacetylation degree were purchased from PolAura (Dywity, Poland). Poly(lactic acid) (1,500,000 g/mol, melting point = 210 ± 10 °C), acetone (analytical grade), NaCl, NH_3_, H_3_PO_4_, Ca(NO_3_)_2_·6H_2_O, NaHCO_3,_ KCl, K_2_HPO_4_·3H_2_O, MgCl_2_·6H_2_O, KOH, HCl, CaCl_2_, chloroauric acid, Na_2_SO_4_, (CH_2_OH)_3_CNH_2_, methanol, ethanol, XTT assay, human osteoscarcoma MG-63 cell line, titanium (IV butoxide), nitric acid, chloroplatinic acid, Dulbecco’s Modified Eagle Medium (DMEM) with high glucose content, phosphate buffer solution (PBS), streptomycin/penicillin, and trypsin were purchased from Sigma-Aldrich, Poznań, Poland. All other reagents were of analytical grade, purchased from Sigma-Aldrich, Poznań, Poland.

### 2.2. Methods

#### 2.2.1. Nanoparticles’ Preparation and Characterization

Briefly, gold nanoparticles were prepared via reduction reaction of chloroauric acid, using sodium citrate at 90 °C and mixed for 1 h. Platinum nanoparticles were obtained by using chloroplatinic acid as a platinum source, which was instilled inside heated up ethylene glycol (150 °C) with constant mixing for two hours, until black color was reached. For the titanium (IV) oxide NPs obtainment, as a precursor, titanium (IV butoxide) was used. To obtain the nanoparticles, the precursor was mixed with ethanol for 30 min, followed by water ethanol solution addition. The ready white precipitate was washed out with distilled water and left to dry. The ready nanoparticles were purified by membrane dialysis, using MWCO 10,000–12,000 Da (Bionovo, Legnica, Poland). For the preparation of hydroxyapatite nanoparticles Ca (NO_3_)_2_·6H_2_O, ammonia and H_3_PO_4_ were used. The ready precipitate was washed out with methanol and calcinated at 250 °C for one hour. The resulted nanoparticles’ polydispersity index (PDI) was calculated, using the Equation (1), given below, based on the TEM images:

PDI = SD/mean(1)

where PDI is polydispersity index, SD is standard deviation, and mean is the arithmetic mean.

#### 2.2.2. Nanofibers Preparation

To obtain a homogenous solution of poly (lactic acid), previously purified polymer was dissolved in the acetone of analytical-grade purity. The solution concentration was 10%. To prepare nanofibers, Industrial Electrospinning System RT Advanced was applied (Linari NanoTech, Pisa, Italy). Nanofibers were prepared at two different potentials, namely 30 and 35 kV. The distance from the spindle was 10. The polymer solution was dosed with the constant speed of 10 mL per hour, with the rotation speed of 1000 RPM. The collector was coated, using aluminum foil. The needle diameter was 500 µm. Obtained nanofibers were left under room temperature, until complete solvent evaporation, and peeled of the foil. Dried nanofibrous mats were used for further investigations.

#### 2.2.3. Chitosan Aerogels Preparation

To prepare an aerogel, for each sample, 0.5 of fungal chitosan was dissolved in aspartic acid water solution, until homogenous solution obtainment. Then, 5 mL of 1,2-propanodiol was added, and the vessel containing polymer was placed inside of the Prolabo Synthewave microwave reactor (Wrocław, Poland). The crosslinking reaction was carried out for 10 min. During the first step, water evaporation was noticed, followed by a crosslinking process. The ready product was washed out from unreacted amino acid residues, until pH = 7. Then, it was mixed with the HA NPs (10 wt %). The scaffolds were freeze-dried. 

#### 2.2.4. Three-Dimensional Scaffolds’ Preparation

The hybrid scaffolds were prepared by placing nanofibrous 3D mat onto chitosan aerogel which contained previously absorbed water (5 mL), followed by lyophilization. The samples were frozen at −20 °C and lyophilized at 0.30 mPa, using ALFA lyophilizator (Donserv, Warszawa, Poland). Then, to embed metallic nanoparticles, 1% methanol solutions containing TiO_2_, Au, and Pt NPs were instilled onto chitosan covered with PLA NFs. The modified biomaterials were left to dry. The chemical composition of the ach sample is given in the Table 1. Each sample contained 0.5 g of the chitosan and 10% *w*/*w* of hydroxyapatite. The concentration of the PLA solution for the fibers’ preparation was 10%. As a solvent, acetone was used.

#### 2.2.5. FT-IR Analysis

Fourier-Transform Infrared Spectroscopy (FT-IR) analysis was performed, using FT-IR Nexus 470 Thermo Nicolet spectrometer obtained from Thermo Fisher Scientific (Waltham, MA, USA). For the measurements, an ATR diamond adapter was used. For FT-IR spectra collecting, each sample was completely dried. 

#### 2.2.6. Liquid-Uptake Ability and Swelling Degree

Liquid-uptake studies were performed in simulated body fluid (SBF). To determine swelling capability, the weighed samples were immersed in SBF for 24 h and weighed again. Based on the weight change, swelling capacity (SC) was determined. To verify the swelling degree, the change in the dimensions of the scaffolds was studied. For this purpose, the samples were cut into cubes (1 cm × 1 cm × 1 cm) and swollen with SBF. After 5 min and 24, their dimensions were measured. Statistical analysis was performed by Excel software, and a *p* < 0.05 value was found to be statistically significant.

#### 2.2.7. XRD Analysis

X-ray powder diffraction (XRD) was carried out, using BRUKER Advanced D8 (Zastávka, Czech Republic). For each analysis, a fully dried sample was used in the amount of 50 mg.

#### 2.2.8. SEM and TEM Analysis

Nanoparticles morphology and diameter were evaluated by Transmission Electron Microscope (TEM), Jeol (Peabody, MA, USA). For the analysis, nanoparticles were dissolved in the methanol of analytical-grade purity, followed by placing onto formvar-coated Cu mesh and left to evaporate under room temperature. The analysis was carried out under HT = 80,000 V, exposure time of 800 ms, and electron (e) dose of 2771.9 e/nm^2^. The nanoparticles’ size was determined by Jeol software. The scaffolds’ components were investigated via an FEI Quanta 650 FEG Scanning Electron Microscope purchased from FEI (ThermoFisher Scientific, Oregon, USA) with HV = 10 kV. The elemental analysis was performed by using an EDAX^®^ adapter (X-ray fluorescence method).

#### 2.2.9. Inorganic Matrix Formation and DC-Induced Inorganic Matrix Formation Study

To determine prepared scaffolds in terms of their positive effect on the inorganic matrix formation, which is an important part of the biomineralization, the scaffolds were placed inside sealed vials containing SBF and stored inside of a CO_2_ incubator, at 37 °C, for 7 days. To evaluate the scaffolds’ potential to electrostimulation of biomineralization process, samples were placed inside cubic glass vessels containing two platinum electrodes 5V (direct current) immersed in SBF. Then, the biomaterials were investigated over coverage with HA crystals, using Atomic Absorption Spectroscopy (AAS) PU-9100x (Philips), as well as SEM and XRF methods. 

#### 2.2.10. Cytotoxicity Study

Cytotoxicity study on a human osteosarcoma cell line MG-63 was carried out, using 2,3-Bis-(2-methoxy-4-nitro5-sulfophenyl)-2H-tetrazolium-5-carboxanilide salt assay (XTT). The salt underwent reduction reaction due to the presence of enzymes coming from metabolically active cells. The cytotoxicity study was carried out according to norm ISO 10993-5 Biological evaluation of medical devices, tests for in vitro cytotoxicity. For the experiments, an MG-63 cell line was used. Bone cells culture was conducted for 7 days, under typical conditions (95% CO_2_, high humidity and 37 °C). The cell culture medium (DMEM) with high glucose content, 2 mM Glutamine supplemented with 10% fetal bovine serum was changed every two days. To perform XTT assay, UV-Vis spectrophotometer Agilent 8453 was applied (Santa Clara, CA, USA).

Statistical analysis was performed by Excel software, and a *p* < 0.05 value was found to be statistically significant.

## 3. Results and Discussion

### 3.1. FT-IR Analysis

**Figure 1:**
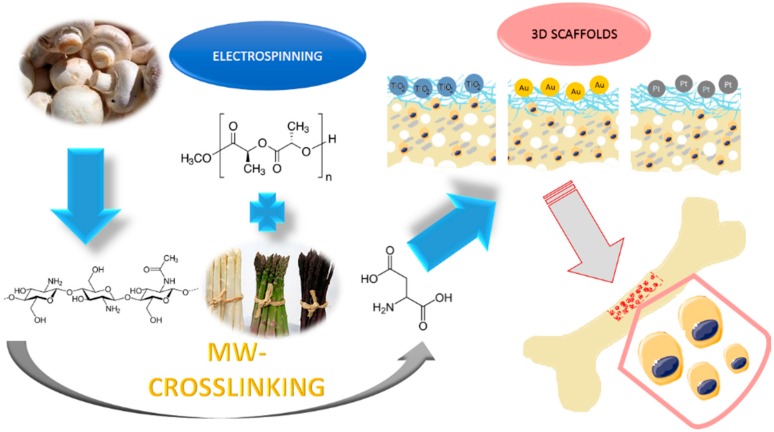
The general 3D scaffolds’ obtainment strategy and application.

The 3D hybrid scaffolds were prepared via combination of PLA nanofibers and chitosan crosslinked aerogel containing hydroxyapatite nanoparticles. The biomaterials were further modified with three types of nanoparticles: TiO_2_, Au, and Pt. The general principle of the supporting matrixes is given in Figure 1. 

**Figure 2:**
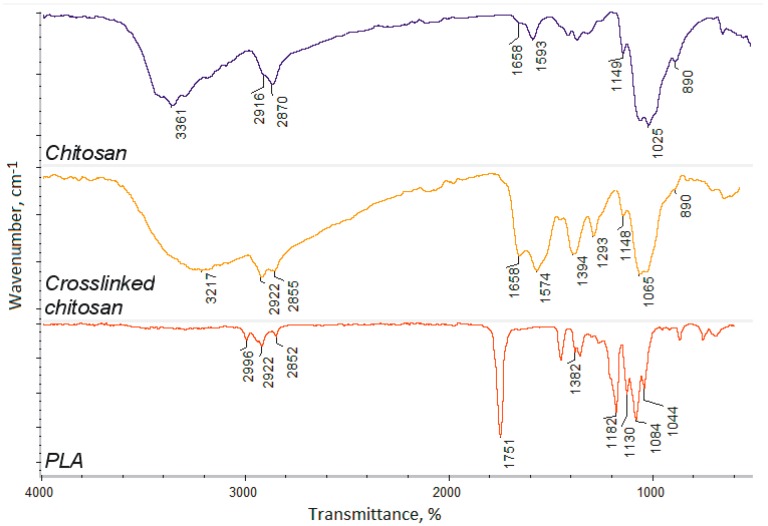
FT-IR spectra of the pure chitosan, crosslinked chitosan, and poly (lactic acid) nanofibers.

The hybrid scaffolds’ components were characterized by FT-IR method (crosslinked chitosan bottom layer and nanofibrous PLA top layer). Figure 2 shows that, during the chitosan crosslinking process, using L-aspartic acid in the 1,2-propanodiol environment, new bonds were formed between free amino groups and carboxyl groups of the amino acid, since the intensity of amide bonds (1658 cm^−1^ for pure chitosan) has significantly increased (1658 cm^−1^ for crosslinked chitosan).

The incorporation of the aspartic acid also proves the increased intensity of free amino groups in the crosslinked polymer (1574 and 1148 cm^−1^), compared to pure chitosan (1593 and 1149 cm^−1^). Such a modification pathway results in the maintenance of free amino groups which are responsible for many positive features and induce cellular responses [20,21,22]. FT-IR spectrum of the nanofibers prepared from the poly (lactic acid) exhibit typical for this polymer band of high intensity at 1751 cm^−1^, and no bands coming from impurities or not-evaporated solvent can be spotted. The other bands come from C–H bending (1382 cm^−1^) and C–O stretching (1182–1044 cm^−1^) [37,38]. What is important is that raw PLA is known for its low bioactivity, as well as hydrophobicity. Thus, application of crosslinked chitosan containing hydrophilic groups can increase cells’ adhesion and cellular responses [15]. Moreover, crosslinked polymer spectrum exhibits a band coming from carboxyl groups at 3217 cm^−1^ that may be present due to the surface degradation as a result of microwave-irradiation, as well as the aspartic acid molecules, which are not fully incorporated into the polymeric matrix only partially grafted leaving one-COOH group free. Superficial degradation of the chitosan causes very slight changes in its chemical structure, as a result of the oxidation of the hydroxyl groups, namely CH_2_OH, which turns into COOH. Due to the presence of the oxygen in the reaction atmosphere, carboxyl groups are being formed, thus replacing hydroxyl ones. No other degradation products are observed. Such chemical structure will provide appropriate chemical conditions for bone-forming hydroxyapatite crystallization and stimulate its nucleation initiation, since polymeric layers will act as an organic template [40,41]. 

**Figure 3:**
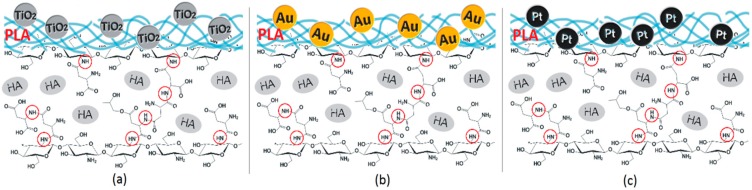
(**a**) 3D hybrid scaffolds’ chemical structure doped with TiO_2_ NPs; (**b**) 3D hybrid scaffolds chemical structure doped with Au NPs; and (**c**) 3D hybrid scaffolds doped with Pt NPs.

Based on the FT-IR data, the proposed chemical structure is given in Figure 3. Such hierarchical composition should well mimic natural bone. Acidic groups provide local charge accumulation, which helps calcium and phosphorous ions’ binding [41]. The chitosan layer mimics the naturally occurring organic bone-forming components on which minerals deposition occurs [40]. Introduced molecules of aspartic acid, as well as poly (lactic acid), enable biological system imitation. Importantly, the chemical structure is rich in both anions and cations, which should help to maintain salts present in the simulated body fluids at elevated level at all times [40]. 

### 3.2. Swelling Properties of the 3D Scaffolds

**Figure 4:**
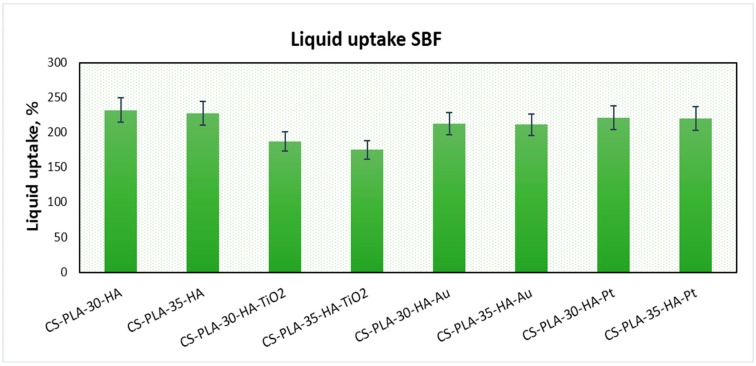
Swelling capacity of the prepared samples.

Hydrogels, which are three-dimensional polymeric structures capable of water solutions sorption, are highly applied in the field of tissue engineering due to their abilities to mimic natural environments. They also have an ability to react on conditions’ changes, such as temperature, pH, or electric field [15]. One of their important parameters is porosity and presence of hydrophilic groups such as amino, hydroxyl, and carboxyl [15], which enables swelling with water and provides space for adhesion and proliferation. Figure 4 presents the results of the swelling-capacity investigations. It can be noticed that all of the prepared samples have excellent swelling capacity.

**Figure 5:**
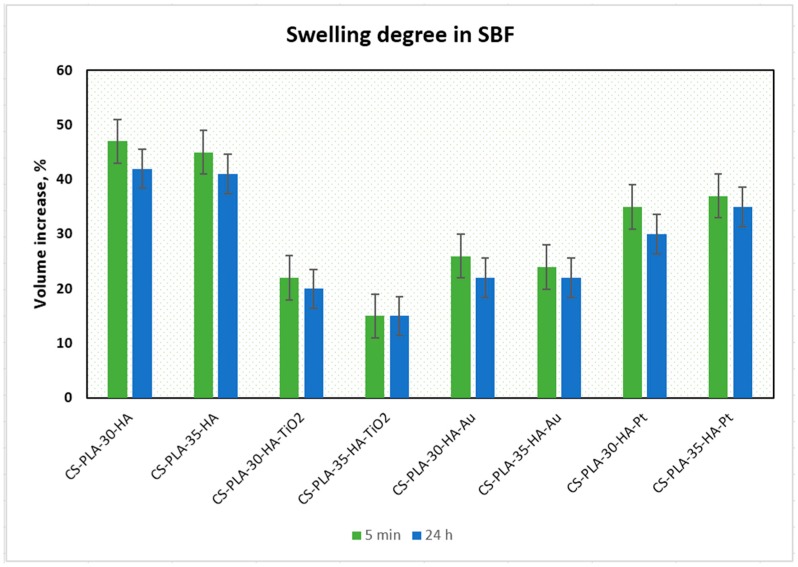
Swelling degree of the prepared samples.

The best results were obtained for samples CS-PLA-30-HA (232%) and CS-PLA-35-HA (227%), which contained only HA NPs. The scaffolds containing metallic NPs were characterized by slightly worse swelling capacity: 221% and 220% for CS-PLA-30-HA-Pt and CS-PLA-35-HA-Pt, respectively, while for CS-PLA-30-HA-Au, it was 213%, and for CS-PLA-35-HA-Au, it was 211%. The significantly lower sorption efficiency was obtained for the samples containing TiO_2_ nanoparticles, and this can be caused by pores’ clogging by agglomerated NPs which hampered water molecules’ migration inside the 3D matrix. What is important is that the studies were performed in the simulated body solution, which contained various ions. Their presence in many cases decreases sorption abilities of the hydrogels due to their interactions with functional groups. In the case of proposed supporting tissue-regeneration materials, all of them exhibited SC above 200%, meaning that they are suitable for bone recovery [1,5,6]. High sorption ability is especially important during the biomineralization process, which significantly hampers water absorption by hydrogels [41]. An excellent swelling capacity also gives them the possibility of incorporation growth factors or genes. Additionally, swelling degree in terms of a scaffold volume change after liquid uptake was investigated (Figure 5). One may observe that all samples, after 5 min, changed their volume, as a result of the soaking up with simulated body fluid. However, the increase was not very high, and this can be explained by the fact that the pores, after contact with water molecules, decreased due to the hydrophilic interactions with free hydroxyl, carboxyl, and amino groups of the scaffold and electrostatic interactions. The swelling degree is correlated with the swelling capacity. Notably, a small change (decrease) of the scaffolds’ volume was observed in time, and the volume stabilized after one hour. Taking everything under consideration, although a small change in the samples’ dimensions was observed, it can be omitted due to the fact that no post-soaking volume increase was observed. The scaffolds are immersed in aquatic solutions (SBF, phosphate buffer, etc.) before implementation inside a patient’s body. If it does not increase its volume in time, it may be assumed that it will not cause any damage to the tissues and nerves placed nearby [15].

### 3.3. XRD Analysis

**Figure 6:**
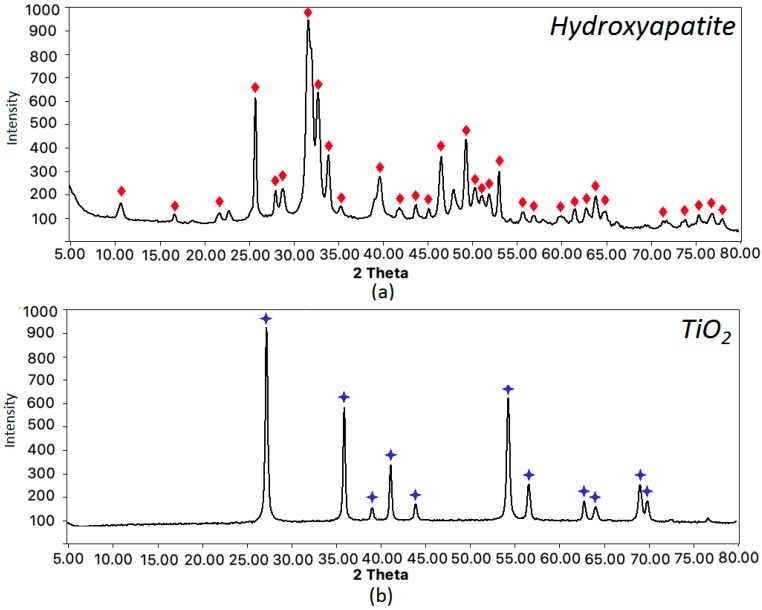
XRD analysis of the scaffolds components: (**a**) hydroxyapatite—the matching patterns are marked with red diamond; (**b**) titanium dioxide—the matching patterns are marked with blue stars.

The scaffolds were prepared by using both organic and inorganic components. Apart from chitosan and PLA, they contained inorganic phase, namely hydroxyapatite. The biomaterials were additionally functionalized by other nanoparticles types, such as titanium dioxide, gold, and platinum. To confirm the crystallinity with ICDD 9–432 of the inorganic components, XRD analysis was carried out for the obtained compounds. The results presented in Figure 6a prove preparation of crystalline hydroxyapatite, whereas Figure 6b shows that prepared metal oxide is TiO_2_ nanoparticles.

### 3.4. Morphology Study of the 3D Scaffolds, and Its Components

**Figure 7:**
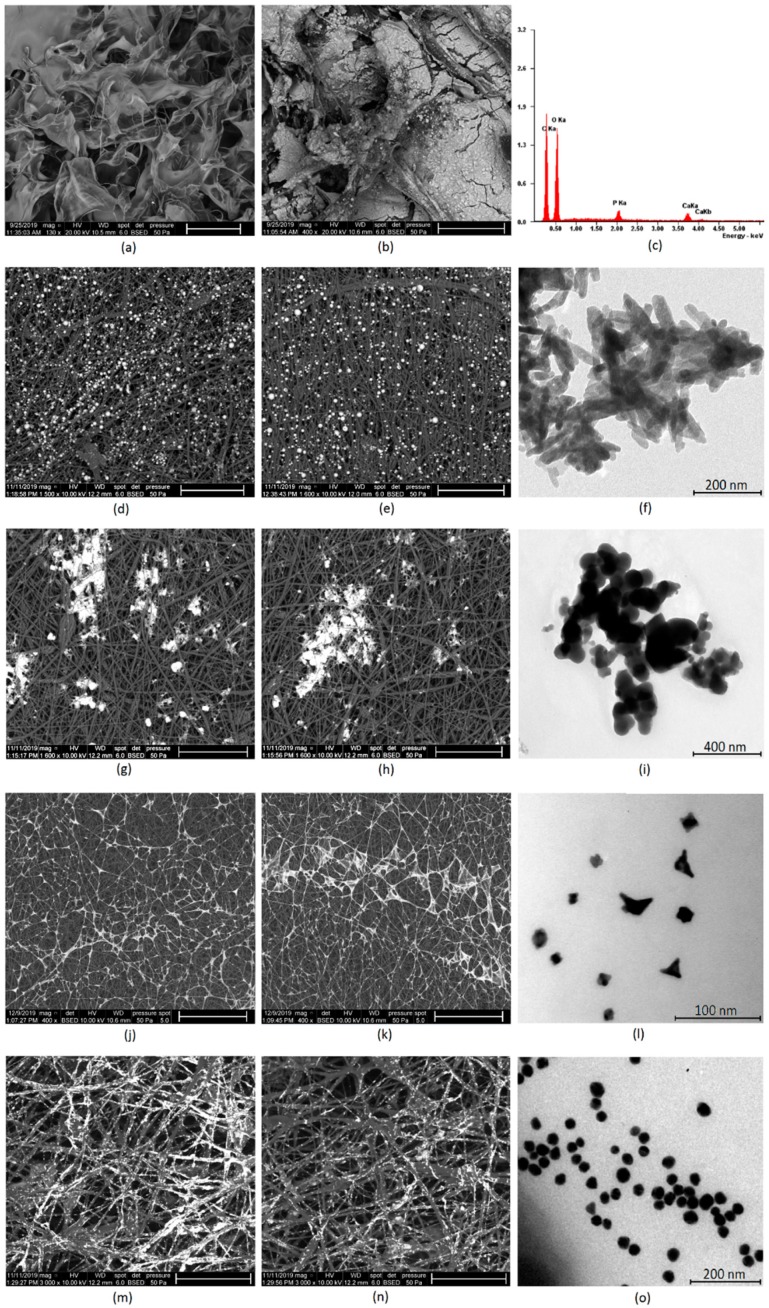
(**a**) SEM microphotograph of the pure chitosan scaffold (scale bar 500 µm); (**b**) SEM microphotograph of the chitosan scaffolds modified with HA nanoparticles (scale bar 200 µm); (**c**) elemental analysis of the scaffold composition; (**d**) SEM microphotograph of the PLA nanofibers obtained under 30kV covered with HA particles (scale bar 50 µm); (**e**) SEM microphotograph of the PLA nanofibers obtained under 35kV covered with HA particles (scale bar 50 µm); (**f**) TEM microphotograph of the prepared HA nanoparticles; (**g**) SEM microphotograph of the PLA nanofibers obtained under 30 kV covered with TiO_2_ nanoparticles (scale bar 50 µm); (**h**) SEM microphotograph of the PLA nanofibers obtained under 35kV covered with TiO_2_ nanoparticles (scale bar 50 µm); (**i**) TEM microphotograph of the prepared TiO_2_ nanoparticles; (**j**) SEM microphotograph of the PLA nanofibers obtained under 30 kV covered with Pt nanoparticles (scale bar 50 µm); (**k**) SEM microphotograph of the PLA nanofibers obtained under 35 kV covered with Pt nanoparticles (scale bar 50 µm); (**l**) TEM microphotograph of the prepared Pt nanoparticles; (**m**) SEM microphotograph of the PLA nanofibers obtained under 30 kV covered with Au nanoparticles (scale bar 30 µm); (**n**) SEM microphotograph of the PLA nanofibers obtained under 35 kV covered with Au nanoparticles (scale bar 30 µm); (**o**) TEM microphotograph of the Au nanoparticles.

To determine morphological properties of the scaffolds, as well as their semi-components, their structure was analyzed by SEM and TEM microscopy. Figure 7a shows pure crosslinked chitosan aerogel, which has a highly porous structure, with pores dimension in the range between 100 and 500 µm, thus providing appropriate conditions for osteoblasts growth, proliferation, and osteogenesis process meeting requirements for bone-tissue engineering [1,5,6]. The scaffold was further modified with synthetic hydroxyapatite, to increase its affinity to this type of tissue and bioactivity (Figure 7b). It can be noticed that HA is well-dispersed on the scaffold surface and does not block the pores. The HA coverage is uniform. Figure 7c shows XRF analysis of the scaffold elemental composition. It can be noticed that the Ca/P ratio is typical for HA (1.6 approximately) and no contaminants such as heavy metal ions are present [40]. The chitosan scaffolds were further modified with PLA nanofibers, to improve its surface properties [2,3,4,5,40]. Figure 7d,e presents the biomaterials surface with incorporated HA nanoneedles, which have a length below 100 nm and width around 20 nm (Figure 7f). The average diameter is 72 nm (length), while the polydispersity index is 0.31. The PLA nanofibers are homogenous and continue. They have coaxial morphology, which means that the fibers have a core–shell structure. The nanofibers obtained under 30 kV are slightly thicker than those prepared under 35 kV. Their density is low enough to provide free diffusion of the gases and nutrients. Their surface is regular. The fiber dimensions are in the range of 0.61–1.56 µm. The average diameter is below 1 µm [2,3,4]. It can be noticed that the nanohydroxyapatite is well dispersed inside the polymeric matrix, in both cases, and are visible in deeper layers. The scaffolds were also functionalized with other nanoparticles, to improve their bioactivity. Figure 7g,h shows biomaterials doped with TiO_2_ NPs, which are known for their biosafety and applicability in bone-tissue regeneration [32,33,34,35]. They have semi-conductive properties. The titanium dioxide particles are present on the nanofibers surface. However, the SEM images show the spot coverage, not a uniform one, and the NPs are partially aggregated due to their hydrophilic nature in contrast to PLA [11]. The nanoparticles are visible only at the top layer of the biomaterial. There are no significant differences between CS-PLA-30-HA-TiO_2_ and CS-PLA-35-HA-TiO_2_ samples. TEM photographs (Figure 7i) present TiO_2_ nanoparticles that seem to have an amorphous structure, round shape, and size below 100 nm. The average diameter is 95 nm, while the PDI is 0.18.

Next, samples were modified with metallic conductive particles (gold and platinum). Figure 7j,k shows CS-PLA-30-HA-Pt and CS-PLA-35-HA-Pt samples doped with Pt nanoparticles. It can be noticed that the NPs are excellently dispersed and exhibit high affinity to PLA fibers. Interconnected fibers covered with conductive platinum particles may provide good current flow. Again, the NPs are present on the superficial fibers. The morphology of the modified fibers (30 and 35 kV) is almost identical. Interestingly, the Pt NPs created a “spiderweb-like” structure. Figure 7l shows platinum nanoparticles. It can be observed that they are of the size between 10 and 20 nm and of various typical for Pt shapes, like rounded, squared, and triangular. The average diameter is 18 nm, while the PDI is 0.28. Finally, Figure 7m,n provides microphotograph of the nanofibers modified with gold nanoparticles. The Au NPs are well dispersed in the polymeric matrix and exhibit very good affinity to poly (lactic acid) fibers. Most of the superficial fibers are almost fully covered with metallic NPs. What is important, on the contrary to both TiO_2_ and Pt NPs, these nanoparticles are present both at the surface of the fibrous layer, as well as in the deeper situated ones. However, in the case of CS-PLA-30-HA-Au sample, the Au NPs are mostly visible at external fibers on the opposite to CS-PLA-35-HA-Au, where gold nanoparticles are located more uniformly. This can be explained by the difference in the fibers’ diameter compared to PLA NFs prepared under 30 kV (wider fibers) and 35 kV (narrower fibers). Such NPs arrangement should provide good current flow and fiber conductivity. Figure 7o shows gold nanoparticles size and morphology. The particles are of a round, uniform shape, 20–40 nm. The average diameter is 35 nm, while the PDI is 0.14. All of the prepared NPs exhibit typical morphology and should not exhibit cell toxicity during direct contact, as they cannot penetrate the cell membrane nor damage it, due to their lack of sharp edges [25,26,27]. Their incorporation should provide extraordinary properties to the hierarchized scaffolds and promote biomineralization process [27,32,33,34,35]. The proposed structure should enable cell adhesion and proliferation, along with osteogenic differentiation, since they meet architectural requirements for bone-tissue regeneration [40,42,43,44,45,46].

### 3.5. Biomineralization Study

The biomineralization process is crucial during bone-tissue regeneration. Thus, scaffolds should not only stimulate osteoblasts’ proliferation but also apatite formation. Mineralization may be induced by the presence of functional groups, such as amino, carboxyl, and hydroxyl, which can bind Ca and P cations [40,41,42]. The results of biomineralization carried out for seven days, under simulated in vitro conditions with and without DC stimulation, are given in Table 2. After one week of bioactivity study, in all samples, mineral sediment was observed. To determine differences between samples, CS-PLA-30-HA (100%) was used as a reference. Standard biomineralization process, which was carried out in simulated body fluid, showed that the addition of TiO_2_ nanoparticles positively affects HA formation, imitating the in vivo process, since the biomineralization of CS-PLA-30-HA-TiO_2_ is higher by 17% compared to CS-PLA-30-HA and by 18% in the case of CS-PLA-35-HA-TiO_2_ compared to CS-PLA-35-HA. In the case of other samples, no superior bioactivity was achieved. There are various methods applied so to accelerate biomineralization, like increased SBF concentration, agitation, or temperature [40]. In this study, an alternative method to enhance biomineralization was proposed based on DC stimulation. The results given in Table 2 show that the application of direct current causes improvement of biomineralization in the case of samples doped with conductive metallic nanoparticles (Pt and Au), while in the case of samples functionalized with TiO_2_ NPs (semiconductor) and HA, no effect was observed. Interestingly, the highest increased of the biomineralization occurred for CS-PLA-30-HA-Au (122%) and CS-PLA-35-HA-Au samples (124%). This can be attributed to the highest conductivity of the gold NPs, as well as the highest coverage of the PLA nanofibers not only on the surface but also on the deeper situated fibers. The improvement in the apatite formation can be explained by the forced ions migration present in SBF (calcium, phosphorous) due to the current flow. Moreover, Au NPs are very well-dispersed, which can lead to the increased number of crystallization nuclei. The fibrous/porous membrane-like structure of the scaffolds causes electroosmosis process, which additionally affects the mineralization process. The results obtained for CS-PLA-30-HA-Pt (116%) and CS-PLA-35-HA-Pt (119%) are slightly worse, and they can be attributed to the lower conductivity of the Pt NPs, as well as their presence only on the external fibers. Finally, it may be noticed that, almost in all cases, the samples prepared using PLA nanofibers obtained under 35 kV, exhibiting better properties in terms of biomineralization than those prepared under 30 kV, which was up to 5% for CS-PLA-30-HA and CS-PLA-35-HA samples.

In both cases, the main process involves nucleation, crystallization, and finally crystals’ growth, which corresponds to in vivo mineralization [40]. The proposed mechanism of the biomineralization on the scaffolds’ surface involves the presence of hydroxyl and carboxyl groups at the mineralization sites coming from *N*-grafted aspartic, as well as degraded chitosan mers inducing apatite nucleation. The first stage occurs due to the calcium ions binding by anionic groups present on the scaffolds, a finding which corresponds to other researchers’ data [36,40,41,42]. 

**Figure 8:**
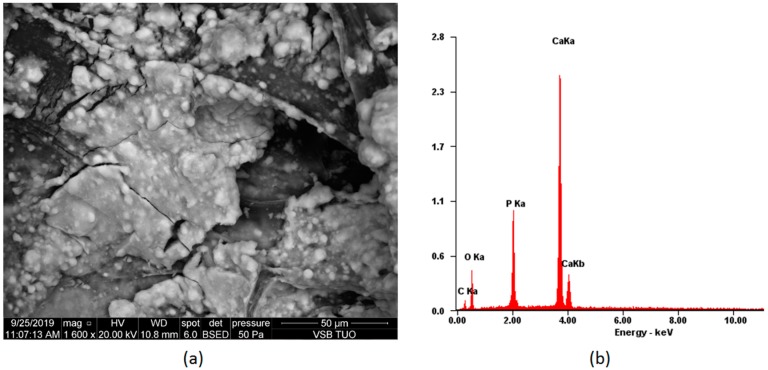
Biomineralization process on the scaffold CS-PLA-30-HA-Au (**a**) SEM microphotograph of the sample; (**b**) elemental composition of the sample.

Figure 8 presents the scaffold surface after seven days of in vitro biomineralization. Sedimented ions, which formed mineral precipitate, can be noticed. The coating is quite smooth and uniform, as is desired [40]. XRF analysis confirmed hydroxyapatite formation. 

### 3.6. Cytotoxicity Study of the Prepared Scaffolds

**Figure 9:**
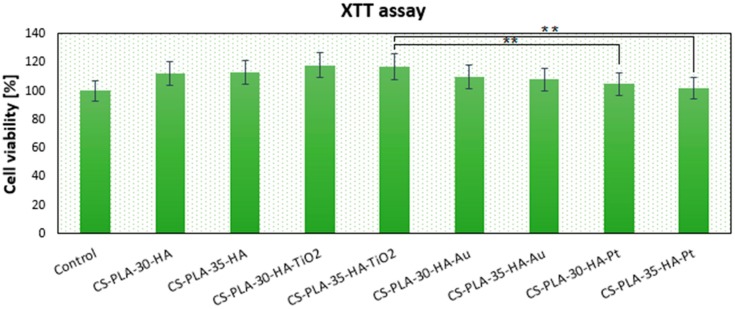
Metabolic activity of osteosarcoma MG-63 cells after seven days of in vitro cell culture determined by XTT assay, (** *p* < 0.01).

The newly developed scaffolds are dedicated to bone-tissue engineering, so one of the principal properties for these biomaterials is biocompatibility. The cytotoxicity study was carried out on an MG-63 human osteosarcoma cell line, which is typically used for this purpose [39]. Figure 9 shows results of cell culture in the presence of scaffolds. After seven days of culture, MG-63 cells’ growth was not disturbed. It can be noticed that, depending on the chemical composition, the number of viable cells is different. Although in all cases the % of living cells is above 100% (control—cells cultured without scaffolds), the presence of various nanoparticles affects osteoblasts’ proliferation activity. Two types of biomaterials display significant bioactivity CS-PLA-30-HA-TiO_2_ (118%) and CS-PLA-35-HA-TiO_2_ (117%). This result corresponds with other researchers’ data since titanium dioxide nanomaterials are known for their biosafety with bone-tissue cells [32,33,34,35]. Slightly worse results were obtained for CS-PLA-30-HA (112%) and CS-PLA-35-HA (113%), and this is not surprising, since the presence of the hydroxyapatite nanoparticles is known to have a positive effect on bone cells [9,27]. Notably, the scaffolds doped with metallic nanoparticles also exhibited good biocompatibility. Gold NPs are well-studied nanomaterials; however, it is known that their in vitro behavior depends on particle size and shape, as well as the presence of stabilizing agents [25,26,27]. Au NPs prepared for this study due to the size above 10 nm do not penetrate nor damage cell membrane and do not affect cell cycles, a finding which corresponds to other researchers’ results [27,29,30]. The results show that, after seven days, the cell number for sample CS-PLA-30-HA-Au was 110% and CS-PLA-35-HA-Au was 108%; thus, their addition did not cause cytotoxic effect. Finally, samples modified with Pt nanoparticles showed 105% and 102% of cells comparing to the reference. Lack of cytotoxicity can be assigned to their size higher than 10 nm and lack of sharp edges. Additionally, in contrast to silver nanoparticles [28], they are characterized by very good chemical stability and are known to disarm reactive oxygen species (ROS), which may have a positive effect on cells’ proliferation, since they prevent their apoptosis triggered by oxygen radicals [25,26,27,31]. Lack of scaffolds’ toxicity can be also assigned to the well-known biosafety of semi-components, such as chitosan [34,35,36] and PLA [2,3,4,5,6,7], which are widely used for bone-tissue regeneration [38]. Moreover, the scaffolds’ architecture seems to provide good conditions for cells’ growth and do not hamper their natural behaviors [38]. The findings show that all of the prepared scaffolds can be considered to be non-toxic and can be used for further studies, which should be focused on in vivo experiments [42,43,44,45,46].

## 4. Conclusions

The aim of the following study was to develop novel 3D bioactive scaffolds with hierarchical structure, using a combination of crosslinked chitosan, electrospinning, and conductive nanoparticles. The innovative scaffolds exhibited extraordinary properties, such as high porosity, excellent swelling properties, and ability of biomineralization electrostimulation. Moreover, the new biomaterials met the requirements for bone-tissue engineering and had a positive impact on MG-63 cells’ proliferation activity. The findings showed that the highest bioactivity in contact with cells exhibited samples modified with nanohydroxyapatite and amorphous titanium dioxide NPs, while scaffolds containing nanogold showed highest positive impact on DC-stimulated in vitro biomineralization. Owing to nanostructured architecture, physicochemical properties, and biocompatibility, the proposed scaffold may play an important role in bone-tissue engineering development.

## Figures and Tables

**Table 1:** Three-Dimensional biomaterials’ composition.

Sample	NPs Type, %	Potential, kV
CS-PLA-30-HA	-	30
CS-PLA-35-HA	-	35
CS-PLA-30-HA-TiO_2_	TiO_2_, 1.0	30
CS-PLA-35-HA-TiO_2_	TiO_2_, 1.0	35
CS-PLA-30-HA-Au	Au, 1.0	30
CS-PLA-35-HA-Au	Au, 1.0	35
CS-PLA-30-HA-Pt	Pt, 1.0	30
CS-PLA-35-HA-Pt	Pt, 1.0	35

**Table 2:** Biomineralization efficiency after seven days of incubation in SBF.

Sample	Standard Biomineralization, %	DC-Induced Biomineralization, %	Ca/P Ratio, -
CS-PLA-30-HA	100	98	1.67
CS-PLA-35-HA	105	99	1.66
CS-PLA-30-HA-TiO_2_	117	99	1.66
CS-PLA-35-HA-TiO_2_	118	99	1.67
CS-PLA-30-HA-Au	100	122	1.70
CS-PLA-35-HA-Au	99	124	1.71
CS-PLA-30-HA-Pt	98	116	1.69
CS-PLA-35-HA-Pt	98	119	1.70
